# A Novel Calibration Method of Line Structured Light Plane Using Spatial Geometry

**DOI:** 10.3390/s23135929

**Published:** 2023-06-26

**Authors:** Huiping Gao, Guili Xu, Zhongchen Ma

**Affiliations:** 1 College of Automation, Nanjing University of Aeronautics and Astronautics, Jiangjun Road, Nanjing 211106, China; 2 School of Computer Science and Communication Engineering, Jiangsu University, Xuefu Road, Zhenjiang 212013, China

**Keywords:** structured light plane calibration, spatial geometry, plane target, least square principle

## Abstract

The line structured light plane calibration method using a plane target cannot produce satisfactory calibration results due to inaccurate positioning of the calibrated points. Field of view noise and sensor noise affect the target light stripe extraction and camera parameter calculation during the calibration process. These factors will cause the calculation of the coordinates of the calibrated point to deviate, and thus affect the light plane calibration. To solve this problem, we propose a new method to calculate the calibrated point based on spatial geometry. Firstly, for the projection line corresponding to the feature point on the light stripe and the corresponding line on the target, a common perpendicular of these two lines above is established, and since the sum of the squares of the distances from the midpoint to the two straight lines is the smallest, the midpoint of the common perpendicular is taken as the calibrated point. Secondly, the target is moved to different positions, and the non-collinear calibrated points are calculated. Finally, the parameters of the light plane are obtained by fitting these calibrated points. This method requires only a checkerboard target, and has a simple calibration process. The experimental results show that the average error of the calibration method proposed in this paper is 0.011 mm, which is less than the 0.031 mm of the calibration method based on the plane target with cross-ratio invariant.

## 1. Introduction

**Figure 1:**
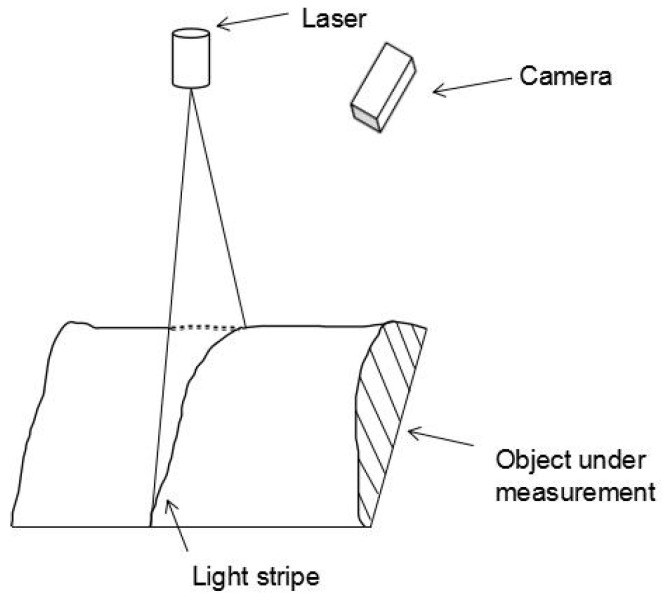
The architecture of a line structured light sensor.

Optical 3D measurement techniques have been some of the most important 3D measuring techniques due to their advantages such as being non-contacting, high precision, fast speed, and so on. Optical 3D measurement techniques can be classified as passive or active based on whether an external light source is used in the measurement system [1]. The active vision technique provides a more accurate result than the passive technique, as it can acquire more information about the shape of the object with the help of an external light source. It has been widely used in many fields, such as reverse engineering [2], industrial inspection [3,4,5,6], 3D reconstruction [7], and robotics [8,9]. According to the light source, the active vision technique can be categorized into: point structured light [10], line structured light, or plane structured light [11]. A line structured light measurement system usually contains a camera and one laser projector. The system projects one laser stripe onto the surface of object, then captures the distorted light stripe modulated by the object surface. Three-dimensional information about the profile of the object can be obtained based on the light stripe center and the system-calibrated results. The architecture of this system is shown in Figure 1. Thus, it can be seen that system calibration is a basic and significant step in the whole measurement process.

Line structured light measurement system calibration includes camera calibration and light plane calibration. Camera calibration is necessary to solve the intrinsic parameters of the camera, and lots of works [12,13,14,15,16] have been conducted on camera calibration. While the present paper concentrates on the calibration of the light plane. According to the shape of the calibration target, the line structured light plane calibration methods can be divided into three-dimensional (3D), two-dimensional (2D), and one-dimensional (1D) methods.

Among 3D light plane calibration methods, Huynh [17] proposed a calibration method with the principle of cross-ratio invariability using a 3D target. The intersection point of the light stripe and the line where the collinear points on the target located were used as calibrated points. Then, the light plane could be obtained by fitting these points. However, the 3D target was required to consist of two or three planes orthogonal to each other, and it was difficult to obtain high-quality images due to the light shielding between planes. Liu et al. [18] proposed a calibration method based on a single ball target. In this method, the coefficients of the light plane equation were obtained by calculating the intersection plane of the sphere target and the cone determined by the light stripe on the ball and the center of the camera. The extraction of spherical contours is easily affected by the environment, and inaccurate contours can further affect the calibration accuracy of the light plane. A movable parallel cylinder target was adopted to calibrate the light plane in [19]. Two ellipses can be obtained from the intersection of the light stripe and the target, and the corresponding equations of the two ellipses and their projected images were established based on the perspective projection transformation. Then, the light plane equation was calculated with the constraint that the minor axis of the ellipse was equal to the diameter of the cylinder. However, the diameter error and the parallelism between the two cylinders would affect the light plane calibration accuracy. Pan et al. [5] proposed a light plane calibration method based on a multi-tooth free-moving target, which can be implemented with a camera equipped with an optical filter. This method took the intersection points of light stripe and multi-tooth target edge as feature points, and compensated the positioning deviation of image feature points based on uncertain models. To calibrate the light plane, this method calculated the coordinates of the feature points in the target coordinate system according to the cross ratio invariance, then combined the camera internal parameters and the Vanishing point of the line where the feature points were located, calculated the coordinates of the feature points in the camera coordinate system according to the camera perspective projection model, and finally fitted the non collinear feature points to obtain the light plane equation parameters. Zhu et al. [20] used a single cylindrical target to calibrate the light plane. The laser projected onto the cylindrical target to form an ellipse. According to the principle of camera perspective projection, the relationship equations were constructed using the geometric characteristics of the ellipse, and the parameters of the light plane can be calculated from the relationship equations. Wu et al. [21] designed a calibration target with a trapezoidal cross-section, and designed a number of characteristic straight lines in the horizontal and vertical directions on its inclined surface. The straight lines can be detected by using the Canny operator and the Hough line detection method, and then the feature points on the target can be obtained. With the angle information between the projected points of the collinear feature points and the optical center of the camera, the coordinates of the feature points in the camera coordinate system can be calculated using the cosine theorem. After that, the coordinates of the feature points on the light stripe can be calculated using cross-ratio invariance. Finally, the parameters of the light plane equation can be obtained by fitting the non-collinear light stripe points.

Wei [22] calibrated the light plane with a 1D target. The intersection point between the light plane and the target was obtained according to the distances between the feature points on the target. The target needed to be moved repeatedly to obtain enough calibrated points for fitting the light plane.

Both 3D and 1D targets need to be designed and manufactured precisely, which is usually expensive. In contrast, a 2D target is often used for camera calibration; its production is mature and accurate. Zhou [23] presented an on-site light plane calibration method using a planar target. The calibrated points are calculated using the principle of cross-ratio invariability as well, and non-collinear points are obtained by moving the planar target repeatedly. Through the conversion relationship between the image coordinate system and the camera coordinate system, Yu et al. [24] solved the equation of the light stripe line in the image in the camera coordinate system, and further calculated the plane equation of the light projection plane, and then calculated the target plane in the camera coordinate system. The intersection of the two planes mentioned above was the equation of the light stripe line on the calibration board in the camera coordinate system. Multiple points on the line were extracted as calibration points, moved the target and repeated the above process to obtain non-common calibration points. These methods based on plane targets are free of expensive equipment, and they are suitable for on-site calibration. However, line structured light plane calibration methods using a plane target fail to obtain satisfactory calibration accuracy due to inaccurate positioning of calibrated points. To obtain more accurate calibrated points, a novel light plane calibration method is proposed in this paper. According to the model of a line structured light vision sensor and the principle of perspective projection, the projection line corresponding to the feature point on the light stripe intersects with the corresponding line on the target in an ideal case. However, field of view noise and sensor noise such as lens distortion, out-of-focus blur, poor laser quality, etc., induce camera calibration error and light stripe extraction error, which lead to the above two calculated lines intersecting in different planes. Based on the spatial geometry observed and the least squares principle, a common perpendicular of the two lines above is established, and since the sum of the squares of the distances from the midpoint to the two straight lines is the smallest, the midpoint of the common perpendicular is taken as the calibrated point. Then, the plane target is moved to different positions to obtain several calibrated points that are not collinear. Finally, the parameters of the light plane are obtained by fitting these points.

The rest of this paper is organized as follows: Section 2 describes the model of the line structured light vision sensor and the proposed line structured light plane calibration method in detail. Section 3 carries out the experiments, and the performance of the presented method is evaluated. Section 4 reaches the conclusion.

## 2. Methods for Light Plane Calibration

### 2.1. Model of the Line Structured Light Vision Sensor

**Figure 2:**
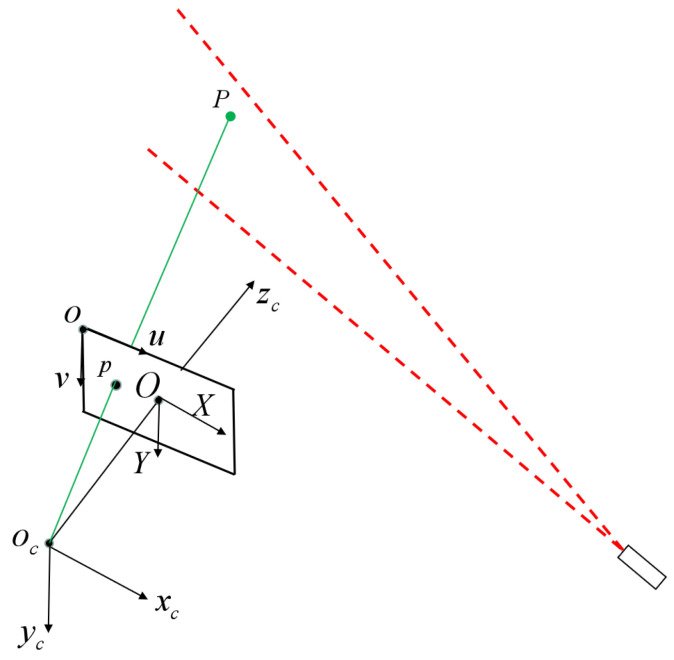
The measurement model of the line structured light vision sensor.

The measurement model of the line structured light vision sensor [25] is displayed in Figure 2. $o_{c}x_{c}y_{c}z_{c}$ is the camera coordinate frame (CCF). ouv is the image coordinate frame in pixels and OXY is the image coordinate frame in millimeters.

Based on the perspective projection model, the equation between the point $P\;=\;{\left(x_{c},y_{c},z_{c}\right)}^{T}$ in the camera coordinate frame and its image coordinate $p={\left(u,v\right)}^{T}$ is obtained as:

(1) $$s\left[\begin{array}{c} u\\ v\\ 1 \end{array}\right]=K\left[\begin{array}{c} x_{c}\\ y_{c}\\ z_{c} \end{array}\right]$$

where $K=\left[\begin{array}{ccc} a_{x} & \gamma & u_{0}\\ 0 & a_{y} & v_{0}\\ 0 & 0 & 1 \end{array}\right]$ is the camera intrinsic parameter matrix obtained by camera calibration. $a_{x}$ and $a_{y}$ denote the effective focal lengths in the *X* and *Y* axes of the image, respectively, $\left(u_{0},v_{0}\right)$ is the principle point, γ is the skew of the two image axes, and *s* is a nonzero scale factor. The light plane equation in the camera coordinate frame can be written as: $a_{c}x_{c}+b_{c}y_{c}+c_{c}z_{c}+d_{c}=0$. The point P locates on this plane, so the mathematical model of the line structured light vision sensor can be expressed as:

(2) $$\left\{\begin{array}{c} s\left[\begin{array}{c} u\\ v\\ 1 \end{array}\right]=K\left[\begin{array}{c} x_{c}\\ y_{c}\\ z_{c} \end{array}\right]\\ a_{c}x_{c}+b_{c}y_{c}+c_{c}z_{c}+d_{c}=0 \end{array}\right.$$

If the camera intrinsic parameter matrix and the parameters of the light plane are known, as well as the image coordinate of the measured point, then the 3D coordinates in the camera coordinate frame of the measured point can be calculated by using Equation (2). In this paper, we assume the camera calibration has been completed [12]; our task is to solve the parameters of the light plane.

### 2.2. The Proposed Line Structured Light Plane Calibration Method

A novel light plane calibration method of free-moving planar targets is proposed in this paper. Firstly, the three-dimensional coordinates of the calibrated point are calculated by using the spatial geometry of the line structured light measurement system as well as least squares principle, and then the parameters of the light plane are obtained by fitting the calibrated points. The steps of light plane calibration are described as follows.

**Figure 3:**
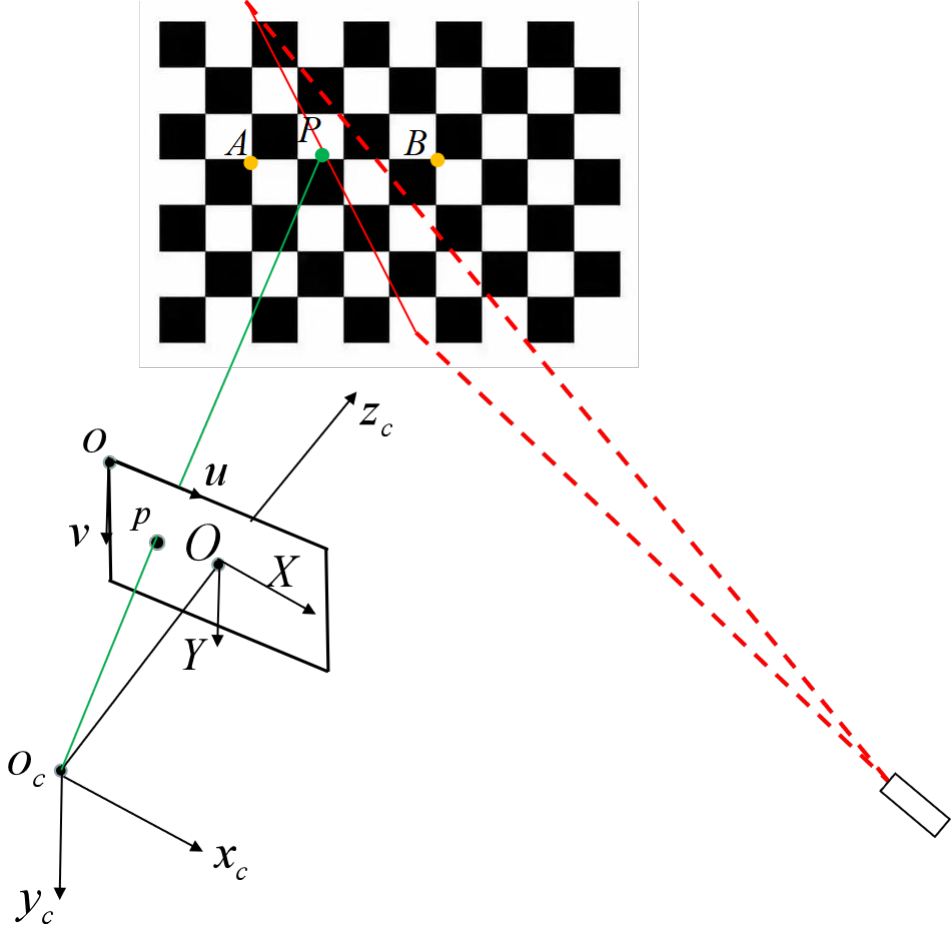
The spatial geometry of the calibrated point for light plane calibration.

As in method [23], the intersection of the light plane and the grid line on the target is used as the calibrated point. According to the model of the line structured light sensor, as shown in Figure 3, the calibrated point *P* is the intersection of the corresponding light projection line $o_{c}-p$ and the corresponding target line A−B without considering errors. However, field of view noise and sensor noise such as lens distortion, out-of-focus blur, poor laser quality, etc., induce camera calibration error and light stripe extraction error, which will result in the lines $o_{c}-p$ and A−B being located on different planes and not having any intersection point. Therefore, combining spatial geometry and the least squares principle, we select the midpoint of the common perpendicular of the lines $o_{c}-p$ and A−B as the calibrated point, and the sum of the squares of the distances from this point to the two straight lines is the smallest.

As shown in Figure 3, the spatial point *P* is the intersection point of the lines $o_{c}-p$ and A−B, and its corresponding projected point is *p*, with image coordinates ${\left[u_{p},v_{p}\right]}^{T}$ in pixels. The coordinates of the point $o_{c}$ are ${\left[0,0,0\right]}^{T}$ in the CCF. As is known, the coordinates of *p* in $z_{c}$ are *f*, which is the focal length of the camera. According to the pin-hole model of the camera, the coordinates of *p* in the CCF can be obtained as:

(3) $$\left[\begin{array}{c} x_{p}\\ y_{p}\\ z_{p} \end{array}\right]=\left[\begin{array}{ccc} d_{X} & 0 & 0\\ 0 & d_{Y} & 0\\ 0 & 0 & 1 \end{array}\right]\left[\begin{array}{c} u_{p}-u_{0}\\ v_{p}-v_{0}\\ f \end{array}\right]$$

where $d_{X}$ and $d_{Y}$ are the sizes of one pixel in the *X* and *Y* axes, respectively.

The coordinates of the points *A* and *B* on the target in the CCF can be given by:

(4) $$\left[\begin{array}{c} x_{i}\\ y_{i}\\ z_{i} \end{array}\right]=\left[\begin{array}{cc} R & T \end{array}\right]\left[\begin{array}{c} LX_{i}\\ LY_{i}\\ 0 \end{array}\right]$$

where i=(A,B), ${\left[LX_{i},LY_{i},0\right]}^{T}$ and ${\left[x_{i},y_{i},z_{i}\right]}^{T}$ are the coordinates of point *A* or *B* in the local world coordinate frame on the target and in the camera coordinate frame, respectively. *R* and *T* are the rotation and translation matrix from the local world coordinate frame to the camera coordinate frame, which can be calculated with Zhang’s method [12].

Since the coordinates of $o_{c}$, *p*, *A*, and *B* in the CCF are calculated, the equations of the spatial straight lines $o_{c}-p$ and A−B are obtained as:

(5) $$f\left(x,y,z\right)=\frac{x-0}{a_{1}}=\frac{y-0}{b_{1}}=\frac{z-0}{c_{1}}$$

(6) $$g\left(x,y,z\right)=\frac{x-x_{a}}{a_{2}}=\frac{y-y_{a}}{b_{2}}=\frac{z-z_{a}}{c_{2}}$$

where $\left[a_{1},b_{1},c_{1}\right]$ and $\left[a_{2},b_{2},c_{2}\right]$ are the directional vectors of $o_{c}-p$ and A−B, respectively, and ${\left[x_{a},y_{a},z_{a}\right]}^{T}$ is the coordinate of point *A* in the CCF.

**Figure 4:**
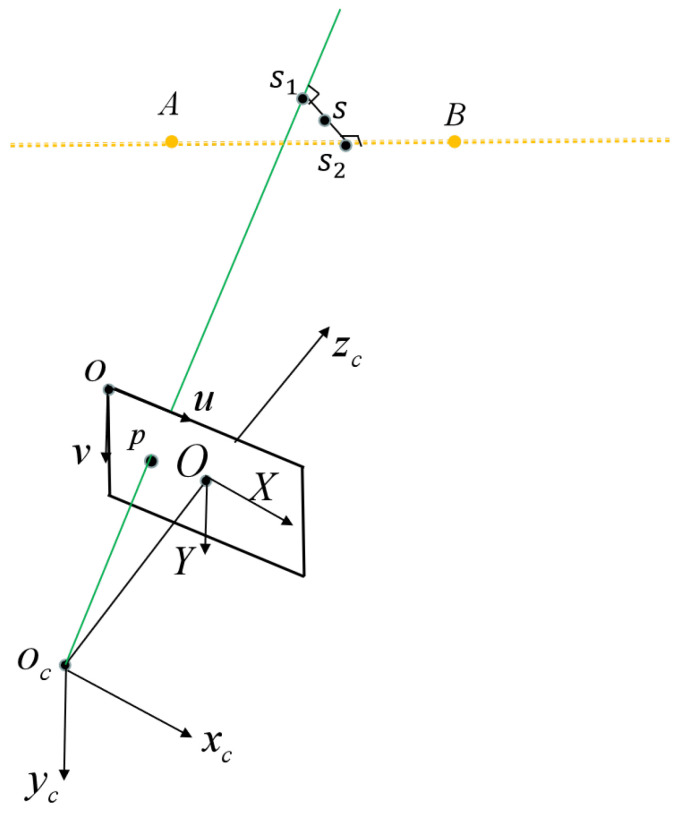
Diagram of calibrated point calculation.

With $f\left(x,y,z\right)$ and $g\left(x,y,z\right)$, we can obtain the common perpendicular of $o_{c}-p$ and A−B, and the common perpendicular intersects with $o_{c}-p$ and A−B at $s_{1}$ and $s_{2}$; we take the midpoint *s* of the line connecting points $s_{1}$ and $s_{2}$ as the calibrated point. A diagram of the calibrated point calculation is shown in Figure 4. Other calibrated points can be obtained similarly: move the target to another different orientation, repeat the above procedures, then, non-collinear calibrated points are obtained.

Finally, the least squares method is used to solve the parameters of the light plane by fitting these calibrated points. The objective function is to minimize the square sum of the distances from the calibrated points to the fitting plane:

(7) $$f\left(a_{c},b_{c},c_{c},d_{c}\right)=\sum_{i=1}^{k}{\left(\frac{\left|a_{c}x_{ci}+b_{c}y_{ci}+c_{c}z_{ci}+d_{c}\right|}{\sqrt{a_{c}^{2}+b_{c}^{2}+c_{c}^{2}}}\right)}^{2}$$

where ${\left[x_{ci},y_{ci},z_{ci}\right]}^{T}$ are the coordinates of the *i* calibrated point in the CCF.

In summary, the procedures for the proposed light plane calibration method are as follows:

(1) Correcting the distortion of the calibration images.
(2) Extracting light stripe centers for all calibration images.
(3) Fitting the line with the extracted light stripe centers.
(4) Fitting the line with the image coordinates of the horizontal collinear points on the checkerboard.
(5) Computing the intersection points of the above two lines on the images.
(6) Computing the line linking the intersection point on the image and the camera optical center in the CCF.
(7) Computing the line where the horizontal collinear points locate on the chessboard in the CCF.
(8) Computing the common perpendicular of the two lines in (6) and (7).
(9) Computing the midpoint of the common perpendicular as calibrated points.
(10) Estimating the equation of the light plane in the CCF by fitting non-collinear calibrated points.

## 3. Experiment Results and Discussion

To verify the feasibility and the effectiveness of the proposed method, we conducted simulated and physical experiments. The simulation experiment is to determine the influence of image noise on the calibration accuracy. The physical experiment is to evaluate the accuracy of our method with Zhou’s method [23] and Yu’s method [24].

### 3.1. Simulation Experiment

The configuration parameters for the simulation experiment are as follows: the camera resolution is 1600 × 1200 pixels, the focal length is 8 mm, and the intrinsic matrix of the camera is

$$K=\left[\begin{array}{ccc} 1000 & 0 & 800\\ 0 & 1000 & 600\\ 0 & 0 & 1 \end{array}\right]$$

the virtual checkerboard is 10 × 7, and the size of each grid is 20 × 20. The equation of the light plane is 1.103x−0.241y−0.856z+390.793=0

The rotation matrixes $R_{1}$ and $R_{2}$ and the translation vectors $t_{1}$ and $t_{2}$ from the virtual target coordinate frame to the camera coordinate frame are:

$$R_{1}=\left[\begin{array}{ccc} 0.9997 & 0.0079 & 0.0232\\ -0.0105 & 0.9936 & 0.1123\\ -0.0222 & -0.1126 & 0.9934 \end{array}\right],\;t_{1}=\left[\begin{array}{c} -10.00\\ 15.00\\ 460.00 \end{array}\right]$$

$$R_{2}=\left[\begin{array}{ccc} 0.9987 & 0.0017 & 0.0503\\ -0.0095 & 0.9878 & 0.1557\\ -0.0494 & -0.1560 & 0.9865 \end{array}\right],\;t_{2}=\left[\begin{array}{c} 10.00\\ -20.00\\ 450.00 \end{array}\right]$$

**Figure 5:**
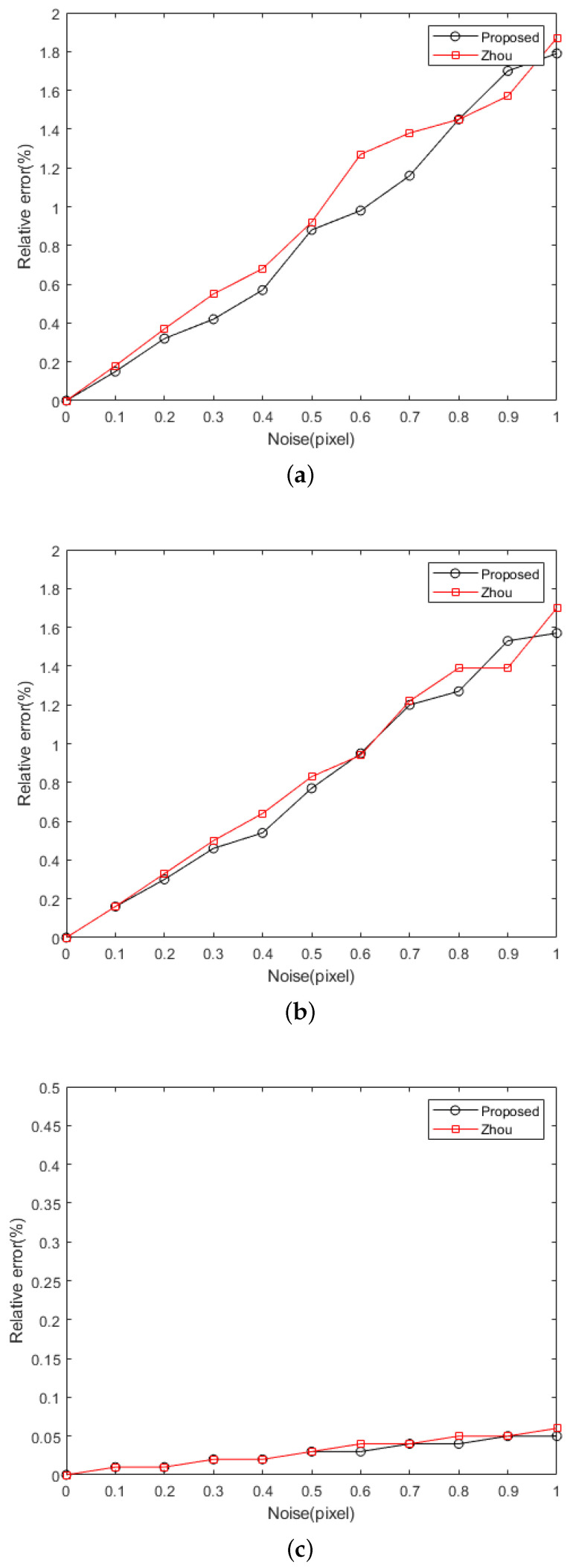
Relative errors of the calibration results at different noise levels. (**a**–**c**) are for plane parameters A, B, and D, respectively.

In the experiment, the target was placed in two different positions. With the preset rotation matrix **R** and the translation vector **t**, the coordinates of the feature points on the target plane in the camera coordinate system can be calculated. By calculating the intersection of the line where the horizontal feature point is located and the preset light plane, the accurate coordinates of the calibrated point in the camera coordinate system can be obtained. Combined with the camera’s internal parameters, the error free projection point coordinates of the calibrated point on the image can be further obtained. In order to verify the influence of the light stripe center extraction error on the light plane calibration accuracy, Gaussian noise with a mean of zero and a standard deviation of 0.1 to 1 pixel with an interval of 0.1 pixels was added to the centers of the light stripe. For each noise level, 100 experiments were carried out to calculate the relative error in the light plane parameters. It should be noted that for the real and calculated light plane equations, we normalized them to Ax+By−z+D=0, and then estimated the relative errors of the plane parameters *A*, *B*, and *D*. The relative errors of the calibration results for our method and Zhou’s method at different noise levels are shown in Figure 5.

As shown in Figure 5, the relative errors increase with the increase in noise for both our method and Zhou’s method [23], and the robustness of the proposed method is comparable to Zhou’s method. Combined with the fact that the extraction accuracy of the light stripe center usually reaches 0.1–0.2 pixels, thus, the relative error in the light plane parameters obtained with our method can reach 0.5% from the simulation results. In this noise range, the method in this paper is slightly better than Zhou’s method.

### 3.2. Physical Experiments

**Figure 6:**
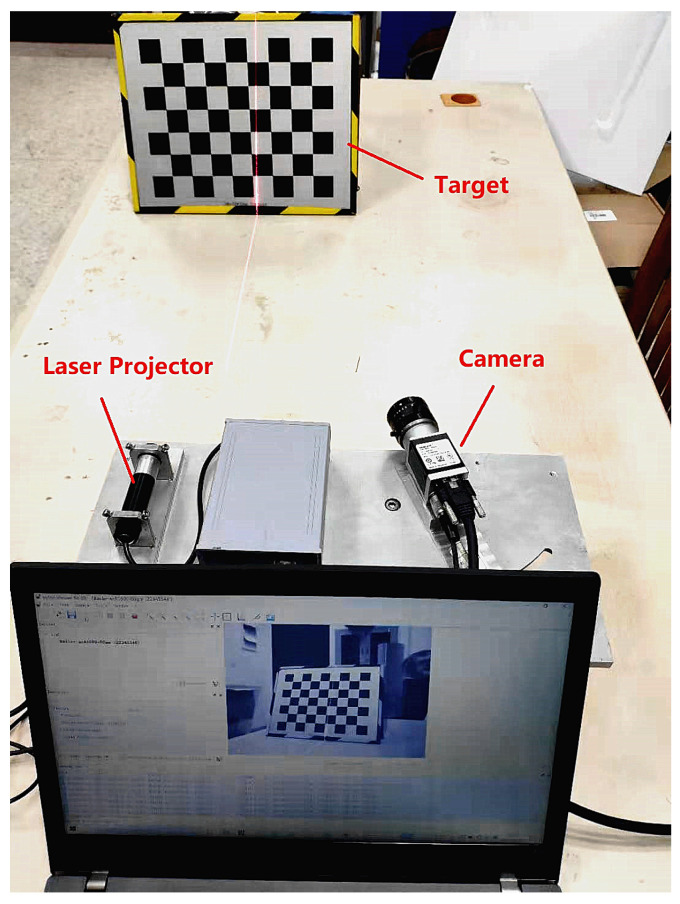
Line structured light vision sensor.

The line structured light vision sensor is composed of a camera with resolution 1600 pixels × 1200 pixels and a single line laser projector with wavelength 650 nm. The calibration equipment is displayed in Figure 6.

The camera intrinsic parameters calibrated with Zhang’s method [12] are as follows:

$$K=\left[\begin{array}{ccc} 1804.75 & 0 & 791.43\\ 0 & 1805.10 & 601.31\\ 0 & 0 & 1 \end{array}\right]$$

The radial distortion coefficients of the camera are $k_{1}=-0.1059$ and $k_{2}=0.1710$.

**Figure 7:**
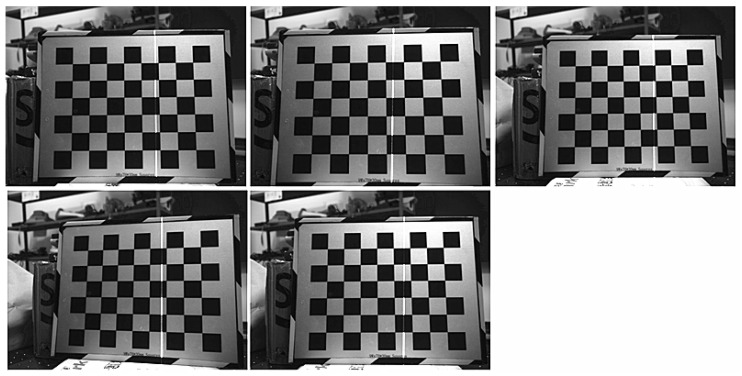
Images used for the line structured light vision sensor calibration.

**Figure 8:**
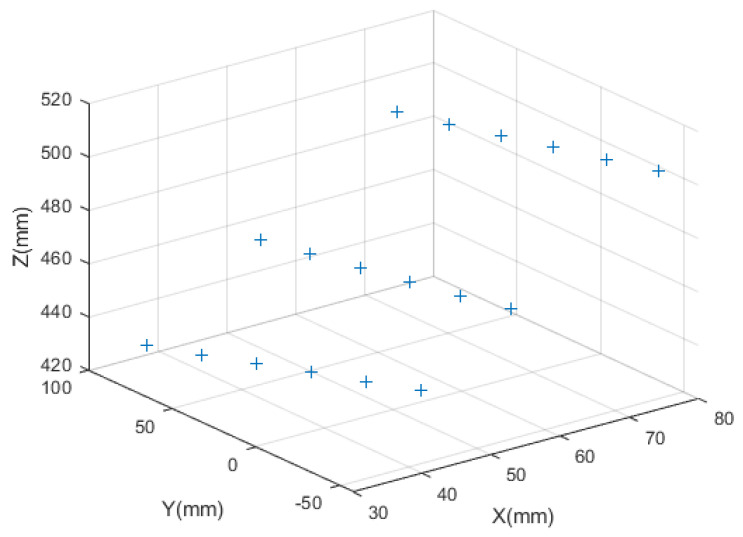
The 3D camera coordinates of the calibrated points on the light plane.

To verify the effectiveness of our method, Zhou’s method [23] and Yu’s method [24] were conducted to compare with the proposed method. A checkerboard was adopted as the target in the light plane calibration experiments, and the spacing of the grid points was 30 mm. We placed the target in front of the line structured light vision sensor five times, and three captured images were used to calibrate the light plane, the other two images were used as test images to verify the accuracy of the calibration results. The images used in the calibration experiments are shown in Figure 7. Firstly, we corrected the distortion of the calibration images, then extracted the coordinates of the light stripe centers and corner points on the undistorted images, and the calibrated points used for fitting the light plane were calculated according to the proposed method and Zhou’s method and Yu’s method. We used 18 calibrated points to calibrate the light plane. The three-dimensional coordinates in the CCF of the calibrated points used in the proposed method are shown in Figure 8, the blue + represents the calibrated points. The calibration results for Zhou’s method, Yu’s method and our method are: 1.724x−0.109y−z+375.073=0, 1.713x−0.111y−z+375.467=0, and 1.727x−0.111y−z+374.997=0, respectively.

For test images, the intersection points of the light plane and the checkerboard in the horizontal direction are called test points. The distance between any two test points calculated with the calibrated line structured light vision sensor was taken as the measured distance dp. The test points in the local world coordinate frame were calculated with the principle of cross-ratio invariance, and they were taken as the approximate ground values. The ideal distance between two test points was taken as dr. The distance deviation was recorded as Δd. The distance between any two test points on the same light stripe is as follows:

(8) $$dr=\sqrt{{\left(x_{w,i}-x_{w,j}\right)}^{2}+{\left(y_{w,i}-y_{w,j}\right)}^{2}+{\left(z_{w,i}-z_{w,j}\right)}^{2}}$$

(9) $$dp=\sqrt{{\left(x_{c,i}-x_{c,j}\right)}^{2}+{\left(y_{c,i}-y_{c,j}\right)}^{2}+{\left(z_{c,i}-z_{c,j}\right)}^{2}}$$

where ${\left[x_{w,i},y_{w,i},z_{w,i}\right]}^{T}$ and ${\left[x_{c,i},y_{c,i},z_{c,i}\right]}^{T}$ are the local world coordinates of the *i*th point and the corresponding 3D camera coordinates.

The distance deviations are shown in Table 1. From Table 1, it can be seen that the RMS error of Zhou’s method is about 0.031 mm, the RMS error of Yu’s method is about 0.075 mm, and that of the proposed method is 0.011 mm. The calibration accuracy of our method is higher than that of Zhou’s method and Yu’s method.

The coordinates of test points in the CCF calculated with Zhou’s method, Yu’s method and the proposed method are shown in Table 2.

## 4. Conclusions

In this paper, a novel light plane calibration method is proposed based on the spatial geometry and the principle of least squares. Our method is validated through simulation experiments and practical experiments. In the simulation experiments, our method is compared with the calibration method [23] based on cross-ratio invariance under different image noise levels, and the results show that our method is robust to noise, and is slightly better than the latter method. In practical experiments, our method is compared with Zhou’s method [23] and Yu’s method [24], and the results show that our method has the highest calibration accuracy. The RMS error of our method is 0.011 mm, which is less than the 0.031 mm of Zhou’s method and 0.075 mm of Yu’s method.

## Figures and Tables

**Table 1:** Accuracy evaluation with distance between two calibrated points.

Position Number	(Point 1 Point 2)	dr (mm)	Zhou’s Method		Yu’s Method		Proposed Method	
			dp (mm)	Δd (mm)	dp (mm)	Δd (mm)	dp (mm)	Δd (mm)
No. 1	(0 1)	30.001	29.980	−0.021	29.978	−0.223	30.011	0.010
	(0 2)	60.000	59.966	−0.034	59.953	−0.047	60.019	0.019
	(0 3)	90.001	89.955	−0.046	89.923	−0.078	90.021	0.020
	(0 4)	120.000	119.949	−0.051	119.891	−0.109	120.019	0.019
	(0 5)	150.000	149.959	−0.041	149.866	−0.134	150.024	0.024
	(1 2)	30.000	29.985	−0.015	29.975	−0.025	30.007	0.007
	(1 3)	60.001	59.974	−0.027	59.945	−0.056	60.009	0.008
	(1 4)	90.001	89.969	−0.032	89.913	−0.088	90.008	0.007
	(1 5)	120.001	119.979	−0.022	119.888	−0.113	120.012	0.011
	(2 3)	30.001	29.989	−0.012	29.970	−0.031	30.002	0.001
	(2 4)	60.002	59.984	−0.018	59.938	−0.064	60.001	−0.001
	(2 5)	90.001	89.993	−0.008	89.913	−0.088	90.005	0.004
	(3 4)	30.009	29.995	−0.014	29.968	−0.041	29.999	−0.010
	(3 5)	60.004	60.004	0.000	59.942	−0.062	60.003	−0.001
	(4 5)	30.000	30.010	0.010	29.974	−0.026	30.004	0.004
No. 2	(0 1)	30.007	29.992	−0.015	29.991	−0.016	30.023	0.016
	(0 2)	60.027	59.990	−0.037	59.980	−0.047	60.043	0.016
	(0 3)	90.037	89.985	−0.052	89.958	−0.079	90.051	0.014
	(0 4)	120.056	119.995	−0.061	119.942	−0.114	120.064	0.008
	(0 5)	150.068	150.022	−0.046	149.936	−0.132	150.086	0.018
	(1 2)	30.022	29.998	−0.024	29.989	−0.033	30.020	−0.002
	(1 3)	60.031	59.993	−0.038	59.967	−0.064	60.028	−0.003
	(1 4)	90.050	90.003	−0.047	89.951	−0.099	90.041	−0.009
	(1 5)	120.062	120.030	−0.032	119.944	−0.118	120.063	0.001
	(2 3)	30.010	29.995	−0.015	29.978	−0.032	30.008	−0.002
	(2 4)	60.028	60.004	−0.024	59.962	−0.066	60.021	−0.007
	(2 5)	90.041	90.031	−0.010	89.955	−0.086	90.043	0.002
	(3 4)	30.020	30.010	−0.010	29.984	−0.036	30.013	−0.007
	(3 5)	60.032	60.037	0.005	59.978	−0.054	60.035	0.003
	(4 5)	30.012	30.027	0.015	29.994	−0.018	30.022	−0.010
RMS error				0.031		0.075		0.011

**Table 2:** 3D coordinates of the test points obtained using different calibration results.

Position Number	Point Index	Zhou’s Method			Proposed Method			Yu’s Method		
		x	y	z	x	y	z	x	y	z
No. 1	1	65.287	−58.002	493.947	65.321	−58.032	494.205	65.252	−57.971	493.680
	2	64.597	−28.357	489.528	64.621	−28.368	489.710	64.553	−28.338	489.197
	3	63.906	1.293	485.109	63.921	1.293	485.216	63.854	1.292	484.714
	4	63.216	30.946	480.689	63.220	30.948	480.723	63.156	30.917	480.233
	5	62.525	60.605	476.269	62.520	60.600	476.231	62.457	60.539	475.751
	6	61.834	90.279	471.846	61.820	90.258	471.737	61.758	90.169	471.269
No. 2	1	63.865	−57.918	491.485	63.897	−57.947	491.737	63.833	−57.889	491.239
	2	63.118	−28.276	486.970	63.141	−28.287	487.147	63.078	−28.259	486.662
	3	62.372	1.371	482.455	62.385	1.371	482.557	62.324	1.370	482.084
	4	61.626	31.014	477.941	61.629	31.016	477.969	61.570	30.986	477.508
	5	60.879	60.673	473.424	60.874	60.667	473.380	60.816	60.610	472.932
	6	60.132	90.348	468.904	60.117	90.326	468.790	60.061	90.242	468.353

## Data Availability

Not applicable.

## Abbreviations

The following abbreviations are used in this manuscript:

CCF	Camera coordinate frame
